# Supplementary material for: Echocardiography and Electrocardiography in Detecting Atrial Cardiomyopathy: A Promising Path to Predicting Cardioembolic Strokes and Atrial Fibrillation
Source: J Clin Med. 2023 Nov 26;12(23):7315. doi: 10.3390/jcm12237315 (PMC10707718; doi:10.3390/jcm12237315)
Supplement: Supplementary file 1 [file jcm-12-07315-s001.zip › jcm-2725359-supplementary.pdf]

Table S1. Variables of the original MVP ECG risk score and probability of AF basis on the punctuation.

P-WAVE VARIABLE	VALUE	SCORE
**Morphology in inferior leads**	Nonbiphasic < 120ms	0
	Nonbiphasic ≥ 120ms	1
	Biphasic	2
**Voltage in lead I**	>0,2 mVv	0
	O,1-0,2 mV	1
	< 0,1mV	2
**Duration**	< 120ms	0
	120-140 ms	1
	>140 ms	2

Probability of AF: . low: 0-1 . intermediate: 3-4 (Odds ratio 2,1) high: 5-6 (Odds ratio 2,4)

Table S2. Normal vàlues and ranges of LA strain components [31].

LA STRAIN COMPONENT	Nº OF STUDIES	MEAN	95% CI	COCHRANE Q
Reservoir	40	39,4	38,0–40,8	1,653 (p < 0,001)
Conduit	14	23,0	20,7–25,2	420 (p < 0,001)
Contractile	18	17,4	16,0–19,0	631 (p < 0,001)

Table S3. Normal and abnormal ranges of 2D-ETT LA indexed biplane volumen [20]

LEFT ATRIUM	NORMAL RANGE	MILDY DILATED	MODERATELY DILATED	SEVERELY DILATED
**Maximum LA volume/BSA (mL/m²)**	16-34	35-41	42-48	> 48
